# MUC5B (rs35705950) Polymorphism and Its Association With Serum Krebs Von Den Lungen-6 (KL-6) and Matrix Metalloproteinase-7 (MMP7) in Patients With Interstitial Lung Disease (ILD) From India

**DOI:** 10.7759/cureus.93801

**Published:** 2025-10-04

**Authors:** Tanya Athavale, Amita U Athavale, Trisha Samant, Tanaya Tipnis, Namrata Neman, Amrutha Jose, Pooja Jaiswal, Anshu Priya, Manisha Madkaikar, Vandana Pradhan

**Affiliations:** 1 Department of Pulmonary Medicine, Environmental Pollution Research Centre, Seth GS Medical College and KEM Hospital, Mumbai, IND; 2 Department of Clinical and Experimental Immunology, Indian Council of Medical Research, National Institute of Immunohematology, Mumbai, IND; 3 Department of Biostatistics, Indian Council of Medical Research, National Institute of Immunohematology, Mumbai, IND; 4 Department of Pediatric Immunology and Leukocyte Biology, Indian Council of Medical Research, National Institute of Immunohematology, Mumbai, IND

**Keywords:** connective tissue disease-associated interstitial lung disease, fibrotic hypersensitivity pneumonitis, genotype-phenotype association, idiopathic pulmonary fibrosis, nonspecific interstitial lung disease (nsip), serum biomarkers, single nucleotide polymorphisms (snps)

## Abstract

Purpose

Krebs von den Lungen-6 (KL-6) and matrix metalloproteinase-7 (MMP7) are established serum biomarkers for interstitial lung disease (ILD). A combination of KL-6 and MMP7 may be a useful screening tool for patients at risk of ILD. However, data on their association with MUC5B promoter polymorphism (rs35705950) in Indian ILD patients are lacking. There is a gap in knowledge of MUC5B genotypes in different subtypes of ILD and the diagnostic cut-offs of serum KL-6 and MMP7 in ILD patients from India. The primary aims of this study were to evaluate the proportion of MUC5B promoter polymorphism in different subtypes of ILD and to establish diagnostic cut-offs for serum KL-6 and MMP7 to distinguish ILD patients from healthy controls. The secondary aim was to investigate whether there was any genotype-phenotype association between the MUC5B genotype and serum biomarker levels.

Methods

This cross-sectional study was conducted in 200 ILD patients from a tertiary care institute in India. Serum KL-6 and MMP7 levels were measured using ELISA. Genotyping for MUC5B (rs35705950) was performed using DNA extracted from peripheral blood leukocytes.

Results

Connective tissue disease-associated interstitial lung disease (CTD-ILD) was the most common ILD subtype (45%). KL-6 levels were higher in idiopathic pulmonary fibrosis (IPF) than fibrotic hypersensitivity pneumonitis (fHP) (105.6 ng/mL vs. 65.87 ng/mL; p=0.0409), in patients who died (109.8 ng/mL vs. 68.6 ng/mL; p=0.022), and in the MUC5B (rs35705950) GT/TT genotype (85.07 ng/mL vs. 65.58 ng/mL; p=0.019). MMP7 levels were higher in IPF than CTD-ILD and fHP (14.01 ng/mL vs. 8.690 ng/mL and 7.970 ng/mL; p=0.0175 and p=0.0118, respectively). The minor MUC5B T allele was more frequent in all ILD subtypes.

Conclusion

This study established an association between MUC5B (rs35705950) promoter polymorphism and higher serum KL-6 levels. The T minor allele had a higher frequency in all ILD subtypes. The study also established cut-offs of serum KL-6 and MMP7 to distinguish between Indian ILD patients and controls.

## Introduction

Interstitial lung diseases (ILDs) are characterized by diffuse inflammation of the lung parenchyma that impairs gas exchange, leading to respiratory failure. The global incidence and prevalence of ILD range from one to 70.1 and 6.27 to 97.9 per 100,000 population, respectively. Dhooria et al. estimated crude ILD incidence and prevalence in India to be 10.1-20.2 and 49.0-98.1 per 100,000 population, respectively [1-3].

Krebs von den Lungen-6 (KL-6) was discovered in 1985 in lung adenocarcinoma cells and was subsequently found to be elevated in various neoplasms. Finding KL-6 in damaged and regenerating alveolar epithelial cells suggested that the extent of alveolar epithelial damage could be estimated with serum KL-6. KL-6, also known as mucin 1, is a high-molecular-weight glycoprotein. A Japanese study found serum KL-6 to be elevated in ILDs. By 1999, Japanese insurance companies were using serum KL-6 as a diagnostic marker for ILDs. This molecule is associated with idiopathic interstitial pneumonias (IIPs), hypersensitivity pneumonitis (HP), connective tissue disease-associated interstitial lung disease (CTD-ILD), pulmonary sarcoidosis, and pulmonary alveolar proteinosis (PAP), and with disease progression and lung function impairment in ILDs [4-6]. Despite extensive global study and commercial availability in laboratories, there is a paucity of data regarding serum KL-6 in Indian ILD patients.

Matrix metalloproteinases (MMPs) are a family of over 25 enzymes that cleave different components of the extracellular matrix (ECM). MMPs also release growth factors sequestered in the ECM in the lung and activate latent transforming growth factor β1 (TGFβ1) [7]. The matrilysin (MMP7) gene is most consistently distinctive between fibrotic and normal lungs; that MMP7-deficient mice were protected from bleomycin-induced lung fibrosis suggests a role for MMP7 in fibrosis [8]. Huh et al. reported increased MMP7 in bronchoalveolar lavage (BAL) fluid and lungs of patients with idiopathic pulmonary fibrosis (IPF). Higher serum MMP7 has been found in IPF compared to other ILDs, as well as in patients with rheumatoid arthritis (RA) with ILD (RA-ILD), and is a marker of progression in IPF and fibrotic hypersensitivity pneumonitis (fHP) [7-9].

Seibold et al. (2011) fine-mapped a risk locus for IIP on the p-terminal of chromosome 11 and identified an association between the MUC5B single-nucleotide polymorphism (SNP) rs35705950 in the promoter of the gene encoding MUC5B and familial IPF [10]. IPF lungs overexpressed MUC5B. Three mechanisms for this association were proposed: excess MUC5B impairs mucosal host defenses, causing excessive injury; aberrant alveolar repair by interfering with the interaction of type II alveolar cells with the underlying matrix or with surfactants; or ectopic production of MUC5B leads to bronchoalveolar unit injury. The MUC5B (rs35705950) promoter polymorphism is associated with IPF, interstitial lung abnormalities (ILA), and interstitial lung changes after COVID-19, and the T minor allele has been shown to slow the progression of disease [11-13]. However, data regarding the association of this MUC5B (rs35705950) promoter polymorphism with other ILDs in India are lacking [10-14].

Serum KL-6, MMP7, and the MUC5B (rs35705950) promoter polymorphism are useful for ILD diagnosis, disease severity, and progression. These biomarkers are widely available due to validated commercial assays and the relative ease of identifying SNPs from peripheral blood. However, the association of these parameters in various ILD subtypes needs to be explored. The primary aims of this cross-sectional study from India were to evaluate the proportion of MUC5B promoter polymorphism in different subtypes of ILD and to establish diagnostic cut-offs for serum KL-6 and MMP7 to distinguish ILD patients from healthy controls. The secondary aim was to investigate whether there was any genotype-phenotype association between the MUC5B genotype and serum biomarker levels.

## Materials and methods

Enrolment of patients

Recruitment of patients for this cross-sectional study was conducted from May 2022 to May 2025. Patients diagnosed with ILD on a clinico-radiological basis were included. All patients had a high-resolution computed tomography (HRCT) of the chest, and the ILD was subclassified on the basis of detailed history, HRCT findings, and autoimmune serology conforming to guidelines and after a multidisciplinary discussion (MDD) [15-17]. Additional procedures, including bronchoscopy and lung biopsy, were performed as required to establish a diagnosis at our institute. The study was approved by the Institutional Ethics Committee. After obtaining informed consent, detailed information regarding presenting symptoms, comorbidities, occupation and exposure history, and medications was recorded in the case record form. Details regarding HRCT pattern and pulmonary function tests were also recorded.

Serum biomarker measurement

The blood collected in a plain vacutainer was centrifuged at 2000 rpm for 15 min. Serum obtained was collected in Eppendorf tubes and stored at -20°C. Serum levels of KL-6 (ng/mL) and MMP7 (ng/mL) were estimated by ELISA using commercially available kits. Autoantibodies such as anti-nuclear antibodies (ANA), anti-dsDNA, and anti-neutrophil cytoplasmic antibodies (ANCA) were detected by indirect immunofluorescence assay (IIFA) using commercial kits (Euroimmun, Germany). ANA specificities were tested using an ANA line blot assay with commercial kits (Euroimmun, Germany).

Identification of single-nucleotide polymorphisms

Blood collected in EDTA vacutainers was used for kit-based DNA extraction. Polymerase chain reaction (PCR)-restriction fragment length polymorphism (RFLP) was used for the detection of MUC5B (rs35705950) promoter polymorphism. Primer design was performed using Primer-BLAST, and the restriction endonuclease for the SNP was selected with NEBcutter [18,19]. Standardization of each PCR to determine the annealing temperature was followed by testing patient and control DNA samples. The RFLP results of MUC5B (rs35705950) promoter polymorphism were further verified using Sanger sequencing.

Statistical methods

Statistical analyses were conducted using RStudio (version 4.3.1, Posit Software, Boston, MA). The normality of data distribution was assessed using the Shapiro-Wilk test. Categorical variables were expressed as counts with percentages. Quantitative variables were expressed as median values with the 25th-75th percentile IQR for skewed distributions and as mean±standard deviation (SD) for normally distributed data. Mann-Whitney U and Kruskal-Wallis tests were used for skewed distributions, and t-test and analysis of variance (ANOVA) were used for normally distributed data to compare continuous variables. A two-tailed P value less than 0.05 was considered statistically significant. The sensitivity and specificity of serum biomarkers were determined through receiver operating characteristic (ROC) analysis. Imputed values were calculated using the median.

While analyzing the MUC5B (rs35705950) SNP to test whether the variant was associated with ILD risk, conformity to the Hardy-Weinberg equilibrium (HWE) was tested using the chi-square and Fisher's exact tests in cases and control subjects. The 1000 Genomes database was used for calculating allele frequency in the control population. The proportions of alleles were reported according to South Asian data [20,21].

## Results

Clinical and radiological characteristics

Two hundred ILD patients and 51 healthy controls were enrolled for serological and genotypic analysis (Table 1). CTD-ILD was the most common diagnosis (90 patients, 45%), followed by fHP (30 patients, 15%), IPF (30 patients, 15%), other ILDs (others; 28 patients, 14%), and fibrotic nonspecific interstitial pneumonitis (fibrotic NSIP; 22 patients, 11%). Among CTD-ILD patients, 30 (33.3%) had RA-ILD, 12 (13.3%) had systemic sclerosis-related interstitial lung disease (SSc-ILD), eight (8.8%) had mixed connective tissue disorder-related interstitial lung disease (MCTD-ILD), three (3.3%) had Sjogren’s syndrome-associated ILD, two (2.2%) had ILD with systemic lupus erythematosus (SLE), one (1.1%) had myositis-associated ILD, one (1.1%) had interstitial pneumonia with autoimmune features (IPAF), and the rest had unclassified CTD-ILD (33 patients, 36.6%). Among patients in the “Others” category, six (21.4%) had non-fHP, three (10.7%) had chronic pulmonary sarcoidosis, seven (25%) had combined fibrosis with emphysema (CPFE), four (14.2%) had occupational ILD (three asbestosis, one silicosis), two (7.1%) had cystic lung disease, one (3.5%) had PAP, one (3.5%) had pulmonary alveolar microlithiasis, and four (14.2%) had pulmonary fibrosis following acute respiratory distress syndrome (one after miliary tuberculosis and three after COVID-19). IPF patients were older (66.97±7.632 vs. 54.07±15.97 years; p=0.0001). One hundred thirty-eight patients (68.5%) were female, but males formed the majority in the IPF (56.6%) and other ILD (53.5%) subtypes. Ten fHP patients (33.3%) and seven CTD-ILD patients (7.7%) reported occupational exposure to pollutants. Seventy patients (35%) had systemic hypertension, 51 (25.5%) had type 2 diabetes mellitus, 13 (6.5%) had ischemic heart disease, three (1.5%) had cerebrovascular accident, and six (3%) had chronic kidney disease. Thirty-five patients (17.5%) had a history of pulmonary tuberculosis. IPF patients had the highest prevalence of at least one comorbidity (70%) and a higher percentage predicted forced vital capacity (FVC) compared to fHP and fibrotic NSIP patients (64.70±17.53 vs. 49.08±16.26, p=0.0052, and 49.38±12.74, p=0.0094). One hundred fifty patients (75%) reported exertional breathlessness, while 118 (59%) had cough. Median symptom duration before presentation was shortest for IPF (7.5 months). Eighty-six patients had a definite/probable usual interstitial pneumonia (UIP) HRCT pattern. Eighteen patients (60%) with RA-ILD and five (41.7%) with SSc-ILD had a UIP pattern. Twenty IPF patients had detectable ANA by IIFA, with 13 at titer 1+ and seven at titer 2+; all had negative ANA blot and no symptoms or other features suggestive of any autoimmune condition.

**Table 1:** Baseline demographic characteristics among ILD patients (n=200). This table summarizes the distribution of key parameters across five ILD subgroups: CTD-ILD, fHP, fibrotic NSIP, IPF, and other ILDs. The mMRC grade refers to the Modified Medical Research Council dyspnea grade. Normally distributed continuous variables are presented as mean±SD, whereas other continuous variables are presented as median with IQR (Q1-Q3) and compared by ANOVA and Kruskal-Wallis tests, respectively. Categorical variables are reported as n (%) and compared using the Chi-square test or Fisher’s exact test. We have reported the F statistic for ANOVA tests, the H statistic for the Kruskal-Wallis test, and the Chi-square statistic in the far-right column, as indicated by the corresponding marks in the column of p-values. ^*^p<0.05 (statistically significant) ^**^p<0.01 (highly significant) ^***^p<0.001 (very highly significant) ^@^One-way ANOVA test ^$^Chi-square test ^+^Fisher’s exact test ^#^Kruskal-Wallis test ILD, interstitial lung diseases; CTD-ILD, connective tissue disease-associated interstitial lung disease; fHP, fibrotic hypersensitivity pneumonitis; NSIP, nonspecific interstitial pneumonia; IPF, idiopathic pulmonary fibrosis; FVC, forced vital capacity; mMRC, modified Medical Research Council dyspnea scale; HRCT, high-resolution computed tomography; UIP, usual interstitial pneumonia

Parameters	All ILDs (n=200)	CTD-ILD (n=90)	fHP (n=30)	Fibrotic NSIP (n=22)	IPF (n=30)	Others (n=28)	P-value	Test statistic
Age, years, mean±SD	52.49±15.08	47.53±14.32	53.73±15.18	56.77±11.50	66.97±7.632	48.11±15.07	<0.001^***,^^@^	12.99
Female, n (%)	137 (68.5)	72 (80)	23 (76.6)	16 (72.7)	13 (43.3)	13 (46.4)	<0.001^***,^^$^	21.753
BMI, kg/m^2^, mean±SD	23.73±5.204	23.59±6.124	23.83±4.315	26.70±3.196	24.03±4.550	21.89±4.583	0.134^@^	1.793
Smoking ever, n (%)	10 (5)	2 (2.2)	1 (3.3)	2 (9)	2 (6.6)	3 (10.7)	0.187^+^	
Wood smoke exposure, n (%)	14 (7)	4 (4.4)	5 (16.6)	1 (4.5)	3 (10)	1 (3.5)	0.184^+^	
Occupational exposure, n (%)	31 (15.5)	7 (7.7)	10 (33.3)	5 (22.7)	7 (23.3)	2 (9)	0.004^**,^^$^	15.1581
Presence of comorbidities, n (%)	98 (49)	35 (38.8)	17 (56.6)	13 (59)	21 (70)	12 (42.8)	0.020^*,^^$^	11.0009
FVC, percent predicted, mean±SD	57.30±19.54	60.24±22.12	49.08±16.26	49.38±12.74	64.70±17.53	57.20±18.03	0.030*,^@^	2.655
Cough, n (%)	118 (59)	46 (51.1)	19 (63.3)	15 (68.1)	20 (66.6)	18 (64.3)	0.379^$^	5.5161
Breathlessness, n (%)	150 (75)	62 (68.9)	24 (80)	14 (63.6)	24 (80)	26 (93)	0.052^$^	8.8696
mMRC grade, mode	2	2	2	2	1	2		
Duration of symptoms, median (Q1-Q3)	12 (11-12)	12 (10-24)	12 (6-24)	24 (6-36)	7.5 (4-12)	12 (4-36)	0.053^#^	9.340
HRCT pattern								
UIP, n (%)	86 (43)	35 (38.8)	8 (26.6)	0	30 (100)	13 (46.4)	<0.001^+^	
NSIP, n (%)	85 (42.5)	50 (55.5)	8 (26.6)	22 (100)	0	5 (17.8)		
Others, n (%)	29 (14.5)	5 (5.5)	14 (46.8)	0	0	10 (35.7)		

Serum biomarkers in healthy controls and interstitial lung disease patients

We analyzed serum MMP7 levels in 187 patients and serum KL-6 levels in 143 patients. Median levels of KL-6 and MMP7 differed between ILD patients and controls (Table 2). ROC curve analyses for these parameters identified the best cut-offs to distinguish ILD cases from healthy controls (Table 2 and Table 3). A cut-off >27.42 ng/mL for serum KL-6 showed sensitivity 87%, specificity 88%, and area under the curve (AUC) 0.8881 in distinguishing ILD patients from controls. A cut-off >3.135 ng/mL for serum MMP7 showed sensitivity 75.94%, specificity 78.43%, and AUC 0.7439 in distinguishing ILD patients from controls.

**Table 2:** Serum biomarkers of ILD patients. All values are expressed as median (IQR). This table presents the serum concentrations of MMP7 and KL-6 across healthy controls (n=51) and patients with various ILD subtypes, including CTD-ILD, fHP, fibrotic NSIP, IPF, and other ILDs. Serum levels of MMP7 were analyzed for 187 patients, and serum KL-6 levels were analyzed for 143 patients. All data are expressed as median values with IQR (Q1-Q3). Statistical comparison was performed between ILD patients and controls using the Mann-Whitney test and within ILD subgroups using the Kruskal-Wallis test. P-values indicate statistical significance between healthy controls and all ILD cases (left column), as well as within ILD subgroups (right column). The U statistic is reported for the Mann-Whitney test, and the H statistic is reported for the Kruskal-Wallis test. ^α^serum MMP7 analysis ^β^serum KL-6 analysis ^&^Mann-Whitney test ^#^Kruskal-Wallis test ILD, interstitial lung diseases; CTD-ILD, connective tissue disease-associated interstitial lung disease; fHP, fibrotic hypersensitivity pneumonitis; NSIP, nonspecific interstitial pneumonia; IPF, idiopathic pulmonary fibrosis; MMP7, matrix metalloproteinase 7; KL-6, Krebs von den Lungen-6

Parameter tested	Healthy controls (n=51)	All ILDs^α, β^	p-value	Test statistic	CTD-ILD	fHP	Fibrotic NSIP	IPF	Others	P-value	Test statistic
MMP7 ng/mL^α^	2.710 (2.130-3.050)	9.250 (3.200-16.48)	<0.001^&^	2443	8.690 (2.420-15.95)	7.970 (2.795-12.64)	11.70 (5.210-26.15)	14.01 (8.593-21.26)	8.105 (2.583-12.39)	0.042^#^	9.872
KL-6 ng/mL^β^	12.07 (8.580-13.82)	72.47 (42.68-120.1)	<0.001^&^	816	74.48 (37.23-117.7)	65.87 (40.48-90.88)	89.26 (48.25-156.2)	105.6 (53.62-194.4)	62.66 (42.68-111.0)	0.196^#^	6.021

**Table 3:** ROC analyses of serum biomarkers in ILD patients versus healthy controls. This presents the diagnostic performance of serum MMP7 and KL-6 levels in distinguishing ILD patients from healthy controls. Statistically significant cut-off values were determined using ROC curve analysis, and the corresponding sensitivity, specificity, AUC, 95% CI, and p-values are reported. These metrics reflect the discriminatory accuracy of each biomarker, with statistical significance assessed using Youden’s Index-optimized thresholds. ILD, interstitial lung disease; MMP7, matrix metalloproteinase 7; KL-6, Krebs von den Lungen-6; ROC, receiver operating characteristic; AUC, area under the curve

Parameter tested	Cut off	Sensitivity (%)	Specificity (%)	AUC	95% CI	P-value
MMP 7 ng/mL	>3.135	75.94	78.43	0.7439	0.6791 to 0.8087	<0.001
KL-6 ng/mL	>27.42	87.41	88.24	0.8881	0.8215 to 0.9548	<0.001

Serum biomarkers in subtypes of interstitial lung disease

KL-6 levels were higher in IPF than in fHP patients (105.6 ng/mL, IQR (53.62-194.4 ng/mL) vs. 65.87 ng/mL, IQR (40.48-90.88 ng/mL), p=0.0409). A serum KL-6 cut-off >89.7 ng/mL differentiated IPF from fHP with 61% sensitivity and 75% specificity (AUC 0.6786, p=0.0406).

Serum MMP7 levels differed between the five ILD subtypes (Kruskal-Wallis test, Table 2). Serum MMP7 levels were higher in IPF patients than in CTD-ILD (14.01 ng/mL, IQR (8.593-21.26 ng/mL) vs. 8.690 ng/mL, IQR (2.420-15.95 ng/mL)) and fHP (7.970 ng/mL, IQR (2.795-12.64 ng/mL); p=0.0175 and p=0.0118). A cut-off >10.72 ng/mL for serum MMP7 distinguished CTD-ILD from IPF with 64% sensitivity and 63% specificity (AUC 0.6494, p=0.0181). A cut-off >9.240 ng/mL distinguished IPF from fHP with 75% sensitivity and 64% specificity (AUC 0.6945, p=0.0125).

MUC5B (rs35705950) single-nucleotide polymorphism

One hundred four controls were included for analysis of the MUC5B promoter polymorphism. The minor T allele was more frequent in the ILD cohort than in controls (19.6% vs 5.8%; odds ratio=3.2; 95% CI, 1.58-6.3; P=0.0008). The MUC5B (rs35705950) polymorphism conformed to the HWE in the controls (P=0.4) but not in the ILD cohort (P=0.0062). The odds ratios for ILD among patients heterozygous (GT) and homozygous (TT) for the risk allele were 4.43 (95% CI, 2.23-8.9; P<0.0001) and 5.9 (95% CI, 0.88-66.22; P=0.008), respectively. The minor T allele was more frequent in all subtypes of ILD compared to controls (Table 4 and Table 5).

**Table 4:** Allele frequencies of MUC5B rs35705950 in ILD patients and controls. This table presents the genotype and allele frequencies of MUC5B rs35705950 in ILD patients and healthy controls. The values are shown as n (%). Statistical comparison between ILD patients and controls was performed using the Chi-square test for both genotypes and allele frequencies. Odds ratios and CI were calculated using regression analysis. The Chi-square statistic is reported for the Chi-square test. ^$^Chi-square test ILD, interstitial lung disease

MUC5B rs35705950	Genotype			P-value	Test statistic	Allele		P-value	Test statistic
All ILD	GG (%)	GT (%)	TT (%)	<0.001^$^		G (%)	T (%)	<0.001^$^	
	126(64.9)	60 (30.9)	8 (4.1)		22.6298	312 (80.4)	76 (19.6)		11.4617
Controls	93 (89.4)	10 (9.6)	1 (0.96)			196 (94.2)	12 (5.8)		
		OR (95% CI)	OR (95% CI)			OR (95% CI)			
		4.43 (2.23-8.9)	5.9 (0.88-66.22)			3.222 (1.588-6.311)			

**Table 5:** MUC5B rs35705950 genotypes among ILD subtypes. This table presents the genotype and allele distribution of the MUC5B promoter polymorphism rs35705950 in healthy controls and patients with various ILD subtypes, including CTD-ILD, fHP, IPF, fibrotic NSIP, and other ILDs. Genotypic frequencies (GG, GT, TT) and allelic frequencies (G, T) are reported as n (%). P-values indicate statistical significance for genotype and allele comparisons between controls and ILD patients, as well as among ILD subtypes, using the Chi-square test and Fisher’s exact test. The test statistic is reported for the Chi-square tests. These results highlight the potential genetic association of rs35705950 with ILD susceptibility and subtype differentiation. ^+^Fisher’s exact test ^$^Chi-square test SNP, single-nucleotide polymorphism; ILD, interstitial lung disease; CTD-ILD, connective tissue disease-associated interstitial lung disease; fHP, fibrotic hypersensitivity pneumonitis; NSIP, nonspecific interstitial pneumonia; IPF, idiopathic pulmonary fibrosis

SNP	Genotype			P-value	Test statistic	Allele		P-value	Test statistic
MUC5B rs35705950	GG (%)	GT (%)	TT (%)			G (%)	T (%)		
All ILD	126(64.9)	60 (30.9)	8 (4.1)	<0.001^$^	22.6298	312 (80.4)	76 (19.6)	0.0008^$^	20.5452
Controls	93 (89.4)	10 (9.6)	1 (1)			196 (94.2)	12 (5.8)		
CTD-ILD	62 (70.5)	23 (26.1)	3 (3.4)	0.002^+^		147 (83.5)	29 (16.5)	0.00^$^	11.4617
fHP	20 (71.4)	5 (17.8)	3 (10.7)	0.012^+^		45 (80.4)	11 (19.6)	0.003^$^	10.678
IPF	13 (44.8)	15 (51.7)	1 (3.4)	<0.001^+^		41 (70.7)	17 (29.3)	<0.001^$^	25.875
Fibrotic NSIP	12 (57.1)	9 (42.9)	0	0.001^+^		21 (70)	9 (30)	0.001^$^	19.1345
Others	19 (67.9)	8 (28.6)	1 (3.5)	0.015^+^		46 (82.1)	10 (17.9)	0.011^$^	8.4396

Mortality of patients during the study period

Twenty-four patients (12%) died during the study period: 8 CTD-ILD (33.4%), 6 fHP (25%), 6 fibrotic NSIP (25%), and 4 IPF (16.6%) patients. Serum KL-6 levels were higher in patients who died (109.8 ng/mL, IQR (57.14-159.4 ng/mL) vs. 68.6 ng/mL, IQR (40.01-112.3 ng/mL); p=0.022)

Serum biomarkers related to genotypes

Serum KL-6 levels were higher in patients with the MUC5B (rs35705950) GT/TT genotype than in those with the GG genotype (85.07 ng/mL, IQR (49.57-144.3 ng/mL) vs. 65.58 ng/mL, IQR (39.41-108.9 ng/mL); p=0.019). Serum MMP7 levels did not differ between genotypes (ng/mL).

## Discussion

Serum KL-6 levels have diagnostic utility and can predict ILD disease outcome [22]. This is the first Indian study to establish a cut-off of serum KL-6 levels to distinguish ILD patients from healthy controls. The present study established a cut-off >24.72 ng/mL for serum KL-6 that distinguished ILD cases from controls with 87% sensitivity and 88% specificity among the Indian population. A note must be made about the units of measurement of serum KL-6. Our study used the units ng/mL. Most of the earlier studies used the units U/mL. Previous studies established this cut-off at 402.5 U/mL with lower sensitivity but higher specificity than the present study [5,22-25].

Serum KL-6 levels were higher in IPF than in fHP patients but not different in CTD-ILD patients in this study. Zuo et al. reported higher serum levels of KL-6 in the CTD-ILD group than in the IPF group. Serum KL-6 indicated the presence of ILD in patients with connective tissue disorders (CTDs) with 76% sensitivity and 89% specificity, according to a meta-analysis by Zhong et al. [22]. Similar to the present study, serum KL-6 distinguished between IPF and fHP [23]. Higher serum KL-6 levels were associated with faster FVC decline and lower survival rates in IPF. Higher KL-6 levels correlated with greater disease severity in patients with systemic sclerosis-related ILD. Higher serum KL-6 levels were also found in ILD patients who died during the study.

The MUC5B (rs35705950) promoter polymorphism has been reported with the presence of the minor T allele as a genetic risk factor for pulmonary fibrosis and has been validated to increase the risk of IPF and RA-ILD [26]. Our study reported the minor T allele in 19.6% of ILD cases compared to 5.8% in healthy controls. Other studies reported a higher proportion of the minor T allele in control groups. The highest percentage of the minor T allele among ILD subtypes was in fibrotic NSIP (30%), followed by 29.3% in IPF, 19.6% in fHP, 17.9% in other ILDs, and 16.5% in CTD-ILDs. The MUC5B (rs35705950) minor T allele is common in ILD, not just in the IPF subtype but also in all other ILD subtypes. To the best of our knowledge, this is the first study demonstrating increased frequency of the minor T allele in the MUC5B (rs35705950) promoter polymorphism in ILDs other than IPF and RA-ILD in India. A recent study by Engel et al. challenged the finding that the MUC5B (rs35705950) promoter polymorphism is more common in RA-ILD and suggested no association with greater ILD risk in other CTDs [27].

A cut-off serum MMP7 level >3.135 ng/mL was found to differentiate between ILD patients and healthy controls in the present study, with slightly lower sensitivity and specificity than serum KL-6. Serum MMP7 distinguished IPF from CTD-ILD and fHP. A cut-off >9.24 ng/mL distinguished IPF from fHP with 75% sensitivity and 64% specificity. Cesar et al. established a cut-off >2.07 ng/mL to distinguish IPF and CTD-ILD patients from healthy controls and reported that CTD-ILD had higher serum MMP7 levels than IPF. Alsobrooks et al. suggested that increased MMP7 levels correlated with an increased risk of one-year progression in patients with IPF [28].

The MUC5B (rs35705950) minor allele T was associated with higher serum KL-6 levels. To the best of our knowledge, this is the first study to find an association between the MUC5B (rs35705950) promoter polymorphism and serum KL-6 levels among Indian ILD patients. It remains unclear whether this is due to pathogenic pathways or merely because the minor T allele and higher serum KL-6 levels are more common in ILD patients. A longitudinal study is needed to explore its diagnostic, prognostic, and therapeutic implications. Although the present study has several limitations, it is a single-center cross-sectional study; it does not adjust for potential confounders with multivariate modeling. Although it suggests association, it cannot conclude causality; the study has established cut-offs for biomarkers and reported genotype-phenotype associations in Indians, a cohort with limited data availability. It establishes the relationship between the MUC5B (rs35705950) minor T allele and serum KL-6 levels, as well as the potential utility of serum MMP7 in distinguishing between ILD subtypes.

## Conclusions

This cross-sectional study established cut-offs of serum KL-6 and serum MMP7 to distinguish ILD patients from healthy controls. It demonstrated the potential of serum MMP7 to differentiate between IPF, CTD-ILD, and fHP. The study showed that serum KL-6 is higher in patients with the MUC5B rs35705950 promoter polymorphism and that the MUC5B minor T allele is common not only in IPF and RA-ILD but in all subtypes of Indian ILD patients. It also found that serum KL-6 is higher in patients who died.
